# Examining the Presence of Border Patrol Agents in Hospitals in South Texas

**DOI:** 10.1007/s10903-025-01673-2

**Published:** 2025-03-05

**Authors:** Christine Crudo Blackburn, Mayra Rico, Lauren Knight, Miryoung Lee, Jessica Hernandez

**Affiliations:** 1 Texas A&M University, College Station, USA; 2 The University of Texas Health Science Center at Houston, Brownsville, USA

**Keywords:** Immigration enforcement, Healthcare access, South Texas, Border Patrol

## Abstract

U.S. policy dictates that immigration enforcement should not take place in or near “sensitive locations,” such as healthcare facilities, but Border Patrol agents are often required to accompany detained border crossers to hospitals for medical care. There is little understanding of the presence of Border Patrol agents within healthcare facilities. This study is the first to examine the presence of Border Patrol agents in healthcare settings and its possible impact on the willingness of undocumented individuals to access healthcare. We conducted interviews with 57 emergency medical services (EMS) personnel working in one county in South Texas. Interviews were held between November 2023– January 2024. Interview transcripts were coded using NVivo 14 and thematic analysis was performed on the transcript data. There is a regular, highly visible Border Patrol presence in healthcare settings in the community of study. In many cases, participants stated that they saw Border Patrol agents every time they brought a patient to the hospital. Hospitals are listed as sensitive locations for immigration enforcement, but policies that require agents to accompany detained border crossers result in a near constant presence of Border Patrol agents in hospitals. The regular, visible presence of Border Patrol agents at hospitals in the study community may deter community residents without legal status from seeking healthcare.

**Supplementary Information:**

The online version contains supplementary material available at 10.1007/s10903-025-01673-2.

## Introduction

There is a historically tenuous relationship between hospitals and immigration enforcement in the U.S. While the U.S. has sanctuary hospitals that have sought to protect undocumented individuals and treat all patients regardless of legal status (i.e., individuals can seek care without enhanced risk of facing immigration-related consequences), hospitals have also played a central role in deportation of undocumented individuals who have sought medical care [1]. For example, in 2008 it was discovered that many U.S. hospitals had contracts with a private company to repatriate patients who came in seeking care, frequently against their will [1]. As an attempt to address some of the issues created by deporting individuals from healthcare settings, as well as address complaints about the trauma of immigration enforcement in places such as schools, the U.S. Department of Homeland Security (DHS) established policies discouraging immigration enforcement in “sensitive locations” [2]. The most recent policy on this topic, *Guidelines for Enforcement Actions in or Near Protected Areas*, was issued in 2021. The memorandum, like sensitive locations policies before it [3], states that the U.S. Immigration and Customs Enforcement (ICE) should not take immigration enforcement actions at or near schools, medical and healthcare facilities, churches, social service establishments, and other locations where essential services are provided [4]. The policies stated in this memorandum are only a suggestion, however, and they are often violated in practice [5].

One of the best-known instances of these types of violations is the 2017 case of Rosa Maria Hernandez in Texas. Rosa Maria was a 10-year-old girl with cerebral palsy who was living undocumented in Laredo and needed gallbladder surgery [6]. En route to the hospital, she was stopped by Border Patrol agents at an interior border checkpoint (IBC) and determined to be undocumented. Border Patrol agents followed her to the hospital, waited at the hospital through her surgery and until discharge, and then took her into custody and placed her in a detention facility [6]. The case grabbed the attention of the nation, but the violation of sensitive locations policy is not uncommon [7]. To date, however, most information about these violations is anecdotal.

Immigration enforcement in sensitive locations, such as hospitals, creates fear among undocumented individuals and makes them hesitant to access healthcare [8, 9]. These enforcement activities also create stress for healthcare providers and make it more difficult for them to provide care to their undocumented patients [10]. In some cases, there is even a perception that healthcare providers are cooperating with ICE [11], raising fears that accessing healthcare will lead to deportation for undocumented patients. Though the literature on these outcomes is limited, there is evidence that the level of local law enforcement affects health outcomes for undocumented individuals because fear often leads to delays in accessing needed healthcare [12–15].

Despite cases like that of Rosa Maria Hernandez and some evidence of the role that hospitals play in the deportation of undocumented patients, there is little understanding of what immigration presence and enforcement in hospital settings looks like in the U.S. With this study, we take the first step in understanding this relationship by examining the presence of Border Patrol agents in the healthcare sector in a border community in South Texas.

## Theoretical Framework

The study was conducted using a phenomenological approach [16]. We examined the lived experiences of EMS personnel in South Texas to understand the presence of Border Patrol agents in hospitals. Since this study was exploratory, as there is no other research of which we are aware that examines the presence of Border Patrol in hospitals, the phenomenological approach allowed the research team to create foundational understanding by capturing EMS personnel perceptions.

## Methods

To examine the presence of Border Patrol agents in the healthcare sector in a border community in South Texas, we conducted 57 in-person interviews with emergency medical service (EMS) providers in one county in South Texas. Interviews were held between November 2023– January 2024. All participants were employed by a city-run fire department or a non-profit EMS provider.

### Ethics Statement

This study was approved as exempt by the appropriate Institutional Review Boards (IRBs).

### Research Team Characteristics

Five research team members (CCB, ML, MR, JH, LK) participated in the study. CCB is a white female in her 30s who has led several qualitative research projects in the community of study. She has extensive experience conducting research on the unique dynamics of the Texas border region and is primarily a qualitative researcher. ML is an Asian female in her 50s who has been conducting research in the study community for several years. MR is a Hispanic female in her 20s who is a native Spanish speaker and has experience conducting workforce development in South Texas. In addition, MR has participated in qualitative data collection and analysis for multiple projects in South Texas led by CCB. JH is a Hispanic female in her 20s, who is a native Spanish speaker and has worked as a graduate research assistant on projects in South Texas led by CCB for the past five years. Her qualitative research experience is bolstered by the fact that she grew up in the study community and is, therefore, very familiar with it. Lastly, LK is a white female in her 20s and an undergraduate student researcher. It is likely that the research team’s familiarity with the community of study encouraged open dialogue and facilitated the depth of the interviews.

### Setting and Process

Interviews were conducted in-person in a one-on-one setting. At each location where interviews took place, the research team was provided with private offices or conference rooms to hold the interviews. This setting allowed participants to have privacy during the interview process and the confidential nature of the interviews allowed them to speak openly. All interviews were conducted in English. Participants were only interviewed once– no repeat interviews were conducted– and all interviews took place while participants were on shift. This meant that participants had their radios on them and turned on during the interviews. An understanding was established before each interview started that participants would leave the interview if they needed to respond to a call, i.e. project participation would not affect their job performance or speed of the response. Three interviews were paused to allow participants to respond to calls and continued later in the day. One interview was discontinued while a participant responded to a call but not resumed later because it was nearing its natural end.

Prior to the interviews, participants were given an informed consent document and a verbal description of the research project. The research team member conducting the interview received verbal consent from the participant, which was recorded on the audio recording. Two to three interviews were conducted simultaneously in different private offices and conference rooms and the research team debriefed after each set of interviews. The focus of the debriefings was to informally determine when data saturation had been reached. With the non-profit EMS company, it was determined that data saturation was reached after 30 interviews. With the city-run EMS provider, it was determined that data saturation was reached after 27 interviews. Meaning saturation– the point at which no new thematic dimensions to codes (i.e., subcodes) are created - was later confirmed during analysis.

### Recruitment and Sampling

Participants for this study were recruited using purposive sampling. Using this sampling method, which focuses on intentionally selecting participants based on their characteristics or experience, we selected participants who were currently working as EMTs or paramedics for the two different types of EMS services in the community of study. In recruiting participants, initial contact was made with the administrator of the EMS company and the fire chief of the city fire department. Both individuals agreed to provide spaces to conduct interviews and to help recruit EMS personnel within their respective departments to participate in the study. Due to the support from the administrator and the fire chief, only 5 individuals with the non-profit company declined to participate and zero individuals with the city-run fire department EMS declined to participate. The following table (Table 1) provides basic demographic information for the individuals in the study.

**Table 1:** Participant demographics

Characteristic	Number of Participants	Percentage of Sample
Sex		
Female	10	17.5%
Male	47	82.5%
Race/Ethnicity		
Hispanic or Latino	56	98%
White	1	2%
Length of Time in Profession		
Less than 2 years	9	16%
2–5 years	7	12%
6–10 years	16	28%
11–15 years	5	9%
16–20 years	5	9%
21 + years	15	26%

### Data Analysis

The study was conceptualized by CCB, and interviews were conducted by CCB, ML, MR, and JH. All interviews were audio recorded for accuracy purposes and audio was transcribed using NVivo Transcription. The transcripts of the interview audio recordings were quality checked by CCB, MR, and LK. The quality check process involved listening to the audio recording while reading through the transcript and correcting any transcription that was not verbatim to the audio. The transcripts were coded by all five members of the research team. At the beginning of the coding process, all members of the research team coded the first transcript and then conducted an inter-coder reliability check. There were few discrepancies in coding, but those that existed were discussed and coding consensus was reached. Following this initial inter-coder reliability check, each team member was given a set of transcripts to code so that there would be two coders for each transcript. Throughout the coding process, inter-coder reliability checks were performed periodically by CCB.

The research team used inductive, latent coding [17] for the analysis because this approach allowed the research team to focus on the implicit rather than explicit meaning of the data. Using latent coding, the research team developed an initial set of parent codes and child nodes related to the experiences of EMS providers in treating and transporting undocumented persons. Following this initial round of coding, a second round of latent coding was conducted to consolidate themes and ensure that theme names accurately represented the data. Based on these rounds of coding, the research team determined that we had reached meaning saturation for the primary themes. Once the analysis was completed, both participating organizations were provided with a summary of the study findings and offered an opportunity to provide feedback.

## Results

During the coding process, the research team determined three themes regarding the involvement of Border Patrol in medical care settings. These were: (1) Border Patrol agents accompany ground ambulance transports of undocumented persons in cases where agents are aware of their legal status, (2) Border Patrol agents are a regular presence in area hospitals, and (3) the presence of Border Patrol agents in healthcare settings may affect health-seeking behavior.

### Theme 1. Border Patrol Agents Accompany Ground Ambulance Transports of Undocumented Persons

Most participants discussed their regular interaction with Border Patrol agents (i.e., consistent presence of agents on-scene when EMS personnel responded to a call) when picking up and transporting an undocumented patient for emergency medical care. Participants said that in most of these instances, it was a Border Patrol agent that had called them, either because the patient had injured themselves while coming into the U.S., had asked for medical assistance once apprehended by Border Patrol, or was displaying signs of illness while in Border Patrol custody. When individuals are in Border Patrol custody and the agent or agents accompany the individual, they sometimes ride inside the ambulance and sometimes will follow behind in their own vehicle. Participants were not sure what determined the way in which Border Patrol agents accompanied the undocumented person, but they noted that when an agent rode inside the ambulance, they often offered to assist EMS personnel with providing care in a way that was appropriate for the agent’s medical training. Because of this, EMS personnel often didn’t mind if an agent accompanied them in the ambulance. Table 2 provides example quotes that show the ways in which Border Patrol is most commonly involved in ambulance transports of undocumented persons.

**Table 2:** Border patrol agents accompany ground ambulance transports

Participant ID	Quote
P15	“Basically, we [EMS] transfer [the patient] to the hospital. Of course, like right after that, I guess law enforcement would advise law enforcement there. Then there would be a Border Patrol agent or someone that sticks with the patients.”
P21	“I know at times if we pick someone up on the levy or at the border wall itself, they’ll send an agent there because at that point, I guess they’re under custody to an extent.”
P22	“Usually they’ll follow us in their vehicles to the hospital and they’ll be there with us.”
P27	“They’re in custody, pretty much. Somebody will go or follow us.”
P33	“Border Patrol comes with us because they’re [the patient] in custody at that point.”

### Theme 2. Border Patrol Agents Are a Regular Presence in Area Hospitals

All participants in the study spoke about the frequency with which they say Border Patrol agents were inside the local hospitals when they were there to offboard patients. Participants said they most often saw Border Patrol agents standing in the hallways outside of hospital rooms. They believed that their presence in the hospital was likely because an individual in their custody required care, and they had to stay with the individual throughout their time in the hospital. Table 3 provides example quotes from participants that relate to the theme of Border Patrol agents’ presence in hospital settings.

**Table 3:** Border patrol agents are often present in hospitals

Participant ID	Quote
P1	“We interact very commonly or very often with the Border Patrol. They’ll be there [at the hospital] with other patients or they’ll be… they’ll follow us, respond with us…So they’re there [at the hospital] pretty often.”
P3	“They’re [Border Patrol] usually in the rooms with a curtain closed and you’ll know it’s them because they’re in uniform and it’s usually about 2 or 3. If it’s Border Patrol, they’re usually with somebody that crossed illegally, jumped the fence. Customs usually has the ones that come through the bridge and they just need medical attention and they’ll take them. But we do see them [Border Patrol agents] in there [the hospital] every day.”
P8	“[You see Border Patrol agents in the hospital] Pretty often. So, you were just walking in there, you know, God forbid, with a family member and you’re taking them for an assessment, you’re probably going to see them. You’re going to see them. You’ll see them standing in the hallway or seen down the hallway next to the room with the patient.”
P9	“[I see Border Patrol agents at the hospital] Almost everyday…it’s pretty often.”
P18	“They [Border Patrol] either they follow us over there, or sometimes we will get there, and they’re standing by there with a patient.”
P45	“Times you walk through the hospitals doing transports and stuff and they’re sitting there either in the room or outside. They are there, just sitting.”
P57	“You’ll see them [Border Patrol agents] there at the hospitals. With the guards and stuff like that.”

### Theme 3. The Presence of Border Patrol Agents in Hospitals May Affect the Health-Seeking Behavior of Undocumented Residents

The final theme was that the presence of Border Patrol agents in hospitals may affect the health-seeking behavior of undocumented residents in the community. Among participants, there was some disagreement about whether the presence of Border Patrol agents had an impact on health-seeking behavior, though more participants stated they believed it did than those who believed it did not. Table 4 provides example quotes of participants perceptions of Border Patrol’s presence in hospitals on health-seeking behavior.

**Table 4:** Effect of border patrol presence on health-seeking behavior

Participant ID	Quote
P1	“I think that especially if they [undocumented person] see, you know, somebody in uniform with the truck there [at the hospital], they’re going to be on edge and they’re just gonna… I would say that if somebody would pull up, they’d probably turn around and be like, ‘No, I’m not going to change my mind.’
P3	“It’s just the beast in [name redacted] is completely different. The community here is very… I don’t want to say that they don’t care, but it just doesn’t really bother them. They’re [undocumented residents] seeking medical attention. They’re going to go regardless. They don’t really look out to, oh why is customs here?…Maybe up north, but around here? No, it doesn’t deter any of our patients.”
P9	“I think it [Border Patrol agents in hospitals] could [deter people from seeking care]. It could play a role because there are some people who are very, like scared about it. But I’ve never seen that happen. I mean, I think maybe they would but, I think would have some effect.”
P10	“I would say that’s definitely a possibility [BP presence impacting people’s willingness to go to the hospital]. So anybody that’s there with Border Patrol already has been detained and they don’t really have the option as far as whether or not they want the treatment, the treatments provided. For their safety and I guess the safety of the organization overall. But I have noticed a hesitation, I guess, just overall to some individuals running into border patrol at restaurants or running into Border Patrol just different areas, just being uncomfortable with them knowing their status.”

## Discussion

Our findings show that Border Patrol agents are regularly present in hospitals within the community of study. Participants said it is common to see them waiting in the hallways, and their presence often means patrol vehicles are parked in front of the hospitals. These findings provide the first research-based evidence of regular Border Patrol presence in a designated sensitive location, as all other discussions of the topic have been anecdotal or in news reports of specific policy violations. These findings create a foundation within the literature to build an understanding of how this immigration enforcement presence in hospitals affects the willingness of undocumented individuals to seek care in those facilities– allowing for the identification of ways that violation of this policy serves as a barrier to healthcare access. While our findings do not speak to the extent to which immigration enforcement actions take place, the presence of Border Patrol agents in the hospital setting may correspond with fear that such actions will occur.

Fear of immigration enforcement in healthcare settings can deter access to healthcare for undocumented persons [8, 9], as well as affect the ways in which providers deliver care to undocumented patients. Previous research has shown that perceived discrimination and citizenship status can affect self-reported health outcomes for immigrants [18] and it is possible that the presence of Border Patrol agents in hospitals brings concerns about immigrant status to the forefront of patients minds when they are seeking care. Based on previous findings from the literature, our findings suggest that the regular of presence of Border Patrol agents in ambulances, hospital hallways, and occasionally exam rooms may create an uncomfortable or high-stress environment for both patients and providers. Additionally, the regular presence of Border Patrol vehicles parked outside of the hospital settings while agents are inside could lead undocumented individuals in need of medical care to leave before even entering the hospital for fear that they will encounter an agent once inside. This could also create challenges for undocumented individuals who are transporting a family member requiring medical attention to the hospital or visiting someone who is hospitalized to provide support. Lastly, it could lead to hesitation to seek care among family members of undocumented individuals out of fear that their possible interaction with Border Patrol agents could expose their undocumented family members. In the case that the presence of Border Patrol agents and vehicles at hospitals could deter undocumented individuals from seeking care, it is possible that they will have worse health outcomes for both acute and chronic conditions due to delays in seeking care or complete care avoidance.

It should be noted, however, that the regular presence of Border Patrol agents in hospitals is the result of the agency providing important and needed medical care for migrants within their custody. U.S. Customs and Border Protection policy states that the agency has a responsibility to conduct an initial health screening, provide regular welfare checks, and to ensure that a detained individual gets appropriate medical care in the detention facility or at a local hospital, when needed [19]. Thus, while their presence likely serves as a barrier to healthcare access for undocumented individuals residing within the community, it also is a marker of facilitating healthcare access for border crossers apprehended at the border. Therefore, our work develops a foundation of understanding of the role of Border Patrol in this context, but further study is needed to fully understand the balance between facilitating care for those in custody and the possible deterrence of care for undocumented individuals in the community. Such an understanding would require conversations with undocumented members of the community to determine whether the presence of agents acts as a barrier and, if so, to what extent.

### Limitations

Our study has a few limitations. The qualitative nature of the study and focus on one community in South Texas means that the findings are not generalizable nationwide or to other border communities within Texas. Additionally, all participants of the study were EMS providers, and the study’s focus was on understanding the extent and regularity of Border Patrol presence in healthcare settings. Since we did not interview any undocumented community members, it is not possible to know the effects that this presence has on health-seeking behavior. This limitation is important because, while we achieved meaning saturation based on information provided by EMS personnel, discussion with healthcare providers and undocumented individuals is necessary to full understanding of the nuances of Border Patrol presence in hospitals and the impact it has on health-seeking behavior.

### New Contribution to the Literature

The development of DHS sensitive locations policy was undertaken with the goal of removing fear and barriers to accessing necessary services. Our findings show that there is a regular and visible immigration enforcement presence in hospitals in the community of study. As this is the first study to document Border Patrol presence in hospitals, more research is needed to fully understand the implications of this presence. The ways in which immigration enforcement presence in hospitals both serves as a barrier to healthcare access for undocumented residents and facilitates healthcare access for migrants in custody is nuanced and requires further examination. Our findings do, however, provide the first set of data demonstrating the disconnect between sensitive locations policy and its application.

## Electronic Supplementary Material

Below is the link to the electronic supplementary material.

Supplementary Material 1

## Data Availability

Data is available upon reasonable request from the corresponding author.
