# Supplementary material for: Examining the Presence of Border Patrol Agents in Hospitals in South Texas
Source: J Immigr Minor Health. 2025 Mar 5;27(3):424–30. doi: 10.1007/s10903-025-01673-2 (PMC12037678; doi:10.1007/s10903-025-01673-2)
Supplement: Supplementary file 1 — Supplementary Material 1 [file 10903_2025_1673_MOESM1_ESM.docx]

**Examples of Questions used during semi-structured interviews**

1. Please tell me how long you have you worked as an EMS professional?
   1. How much of that time has been in your current department?
2. How often do you encounter Border Patrol agents during your workday?
   1. Could you describe a normal (average) encounter?
3. How would you describe the relationship between Border Patrol and EMS?
4. Do you see Border Patrol agents in the hospitals when you offboard patients?
   1. How often?
5. Have patients ever changed their mind about wanting to go to the hospital if they see a Border Patrol agent or vehicle outside when you arrive?
   1. Please describe this.
